# Perspectives on immunotherapy via oncolytic viruses

**DOI:** 10.1186/s13027-018-0218-1

**Published:** 2019-02-11

**Authors:** Alberto Reale, Adriana Vitiello, Valeria Conciatori, Cristina Parolin, Arianna Calistri, Giorgio Palù

**Affiliations:** Department of Molecular Medicine, University of Padua, Via A. Gabelli, 63, 35121 Padua, Italy

**Keywords:** Oncolytic virus, Oncolytic virotherapy, Cancer immunotherapy, Cancer gene therapy, Oncolytic HSV-1, Tumor microenvironment

## Abstract

**Background:**

With few exceptions, current chemotherapy and radiotherapy protocols only obtain a slightly prolonged survival with severe adverse effects in patients with advanced solid tumors. In particular, most solid malignancies not amenable to radical surgery still carry a dismal prognosis, which unfortunately is also the case for relapsing disease after surgery. Even though targeted therapies obtained good results, clinical experience showed that tumors eventually develop resistance. On the other hand, earlier attempts of cancer immunotherapy failed to show consistent efficacy. More recently, a deeper knowledge of immunosuppression in the tumor microenvironment (TME) allowed the development of effective drugs: in particular, monoclonal antibodies targeting the so-called immune checkpoint molecules yielded striking and lasting effects in some tumors. Unfortunately, these monoclonal antibodies are not effective in a majority of patients and are ineffective in several solid malignancies. Furthermore, due to their mechanism of action, checkpoint inhibitors often elicit autoimmune-like disease.

**Main body:**

The use of viruses as oncolytic agents (OVs) was considered in the past, while only recently OVs revealed a connection with immunotherapy. However, their antitumoral potential has remained largely unexplored, due to safety concerns and some limitations in the techniques to manipulate viruses. OV research was recently revived by a better knowledge of viral/cancer biology and advances in the methodologies to delete virulence/immune-escape related genes from even complex viral genomes or “to arm” OVs with appropriate transgenes. Recently, the first oncolytic virus, the HSV-1 based Talimogene Laherparepvec (T-VEC), was approved for the treatment of non-resectable melanoma in USA and Europe.

**Conclusion:**

OVs have the potential to become powerful agents of cancer immune and gene therapy. Indeed, in addition to their selective killing activity, they can act as versatile gene expression platforms for the delivery of therapeutic genes. This is particularly true for viruses with a large DNA genome, that can be manipulated to address the multiple immunosuppressive features of the TME. This review will focus on the open issues, on the most promising lines of research in the OV field and, more in general, on how OVs could be improved to achieve real clinical breakthroughs in cancers that are usually difficult to treat by immunotherapy.

## Background

The pharmacological therapy of cancer represents one of the greatest challenges for contemporary medicine. State-of-the-art chemotherapy and radiotherapy protocols can be curative in some hematologic malignancies, such as Hodgkin lymphoma and acute lymphoid leukemia (ALL), and can be successfully combined with other therapeutic solutions like autologous stem cell transplantation [1, 2]. Targeted therapies have also emerged that changed the natural course of diseases like chronic myeloid leukemia or promyelocytic myeloid leukemia [3, 4]. Even for ALL resistant to current therapies, the use of chimeric antigen receptor (CAR)-T cellular therapy provided a major breakthrough [5].

The situation is much bleaker for non-hematologic neoplasms. With very few exceptions, in this case, the hope of a cure rests mainly on the possibility of a radical surgical excision at the moment of diagnosis. If this is not possible, due to extensive local invasion or metastatic dissemination, prognosis remains dismal [6, 7]. Great expectations were associated with targeted therapies, such as small molecule tyrosine kinase inhibitors (TKIs) or monoclonal antibodies directed against receptors overexpressed by cancer cells. Even though these approaches obtained good results in selected patients, in terms of prolonged survival, with a good toxicity profile, it soon became evident that tumors usually develop resistance [8, 9].

Another possible therapeutic strategy is immunotherapy. Although it has been known for quite a long time that the immune system can recognize and kill cancer cells, previous attempts of immunotherapy based on the administration of recombinant cytokines, anti-cancer vaccines or in vitro expanded tumor infiltrating lymphocytes (TILs) did not provide enough efficacy [10, 11]. Still, there were some remarkable exceptions, as a small subset of metastatic melanoma and of clear cell renal carcinoma patients showed long-term remissions after treatment with high doses of recombinant interleukin 2 (rIL-2) [12]. In recent years, new light was shed on mechanisms involved in cancer immunology, and, especially, on the immunosuppressive features of the tumor microenvironment (TME), which mediate escape from tumoricidal immune responses. In particular, cancer has the ability to exploit mechanisms involved in the maintenance of immune peripheral tolerance, either i) directly, by expressing immune checkpoint molecule ligands which dampen the activity of cytotoxic T cells, such as Programmed Death Ligand-1 (PDL-1), or ii) indirectly, by recruiting immune cells with immunosuppressive features, such as CD4+ CD25+ Foxp3+ T regulatory cells (Tregs), immature myeloid-derived suppressor cells (MDSCs), or M2 macrophages [13, 14]. These cells usually express checkpoint molecule ligands and secrete soluble cytokines (e.g. IL-10) or enzymes (arginase and IDO) that hinder cytotoxic T responses. These and other actors, like cancer associated fibroblasts and downregulation of MHC class I molecules by cancer cells, are probably playing a role in TME immunosuppression. Based on these considerations, new cancer immunotherapies were developed, based on checkpoint inhibition by means of monoclonal antibodies directed against Cytotoxic T Lymphocyte Antigen 4 (CTLA-4), Programmed Death-1 (PD-1), or its ligands PDL-1 and PDL-2 [15]. Anti-CTLA-4 humanized antibodies, as ipilimumab, were the first to show therapeutic efficacy against melanoma [16]. On the other hand, anti-PD1 and anti-PDL1 mAbs seem to have a broader spectrum of action (including NSCLC and possibly small subsets of pancreatic and breast cancer), while triggering less autoimmune toxicity [17]. However, also in cancer types considered susceptible to checkpoint inhibitors, more than 50% of patients fail to respond to treatment. In this context, the combination of different checkpoint inhibitors (anti-CTLA-4 and anti-PD1 Abs) yielded better results in melanoma patients, but with increased toxicity [18].

Oncolytic viruses (OVs) are defined as viruses able to selectively replicate in and kill cancer cells [19]. The history of OVs is quite long, since already at the beginning of the twentieth century physicians observed that cancer patients experienced partial disease remissions after natural infections [20]. It was, therefore, hypothesized that cancer cells were somehow more vulnerable to viral infections, and that attenuated viral strains could be used in cancer therapy. However, many factors, including safety concerns, the development of cytotoxic chemotherapy, and the lack of tools to manipulate viruses, hindered research in this field. In recent years OV studies were revived by better knowledge of viral gene function and advancements in molecular biology, which allow precise modifications of viral genomes to maximize both efficacy and safety. Over the last years a new paradigm emerged according to which OVs might also function as a form of immunotherapy [21]. Indeed, it has been shown that the proinflammatory stimuli provided by viruses can overcome the TME immunosuppression and, thereby, elicit a systemic antitumoral immune response. Such a response was observed also when OVs were injected locally (intratumoral injection), rather than systemically [22]. It was demonstrated that the first OV approved for cancer treatment in North America and Europe, the HSV-1 based talimogene laherparepvec (T-VEC), has an immunological mechanism of action, which also causes the regression of uninjected and uninfected metastases [23].

Nevertheless, OVs are still not powerful enough, especially for scarcely immunogenic or immunosuppressive solid tumors, which unfortunately are quite frequent in the population, like pancreatic adenocarcinoma, triple negative breast cancer, hepatocellular carcinoma [24–26]. This lack of efficacy is somehow unexpected, as OVs should make the TME significantly more immunogenic due to inflammation and the presence of viral antigens. Such a consideration fuels the feeling that major improvements in the OV therapy field are at hand.

This review will focus on open issues regarding OVs, and especially their interaction with the TME and the host immune system. The answer to these questions will probably be crucial to fully exploit the therapeutic potential of OVs.

## Main text

As explained above, OVs are emerging as a new, promising form of immunotherapy. In recent years a remarkable array of different OVs has been tested in preclinical cancer models or in phase I/II clinical trials [27]. This plethora of viruses includes, among the others, attenuated strains of human pathogens, such as adenoviruses (AdVs) [28], herpes simplex type 1 (HSV-1) [29], vaccinia virus (VACV) [30], measles [31], mumps virus [32] and influenza A virus [33], or viruses that are naturally poorly pathogenic for humans, including the orthoreovirus strain T3D [34], Newcastle Disease Virus (NDV) [35], vesicular stomatitis virus (VSV) [36], Maraba Virus [37], the rodent H-1 parvovirus [38] and the picornavirus Mengovirus [39], a long list far from being complete [Table 1]. Perhaps the most striking common feature of these heterogeneous OVs is their outstanding safety profile. Indeed, severe adverse effects were very rare and it was unusual that therapy had to be discontinued due to toxicity [40]. Unfortunately, safety was not always matched by efficacy, and so far only the HSV-1 based talimogene laherparepvec was effective enough to be authorized for routine clinical use. Also, efficacy was markedly higher in immunogenic tumors such as melanoma [41].

**Table 1:** A necessarily incomplete overview of currently investigated or clinically available oncolytic viruses

	Adenoviruses (AdVs)	Herpes simplex virus- 1 (HSV-1)	H-1 Parvovirus	Vaccinia virus (VACV)	Measles virus (MeV)	Maraba virus (MARV)	Orthoreovirus (T3D)
Family	*Adenoviridae*	*Herpesviridae*	*Parvoviridae*	*Poxviridae*	*Paramyxoviridae*	*Rhabdoviridae*	*Reoviridae*
Nucleic acid	dsDNA	dsDNA	ssDNA	dsDNA	ssRNA, negative sense	ssRNA, negative sense	dsRNA, segmented
Genome length	~ 30–35 kb	~ 150 kb	~ 5 kb	~ 190 kb	~ 15–16 kb	~ 11 kb	~ 23 kb
Wild-type virus associated diseases	Depending on serotypes, common cause of mild community-acquired respiratory, ocular, gastrointestinal infections.	Immunocompetent host: primary gingivostomatitis or genital lesions, reactivation from latency (cold sores). Occasionally encephalitis. Immunocompromised host: disseminated disease, multiorgan involvement.	Rodent virus. No disease in humans during clinical trials.	Strains derived from animal poxviruses, used as smallpox vaccine. Usually mild local reaction at the site of inoculation. In immunocompromised hosts severe, progressive disease (vaccinia necrosum).	Measles. Severe complications include giant cell pneumonia, subacute sclerosing panencephalitis (SSPE).	Virus isolated from a brazilian sandfly. Limited evidence of natural infection in humans.	Infection usually asymptomatic.
Available therapy	Live vaccine employed by the US army. No established therapy	Highly effective nucleoside analogues (acyclovir, famciclovir, penciclovir, etc.).	None	Disease rare due to smallpox vaccine programs interruption. Cidofovir possibly active.	Measles-mumps-rubella (MMR) vaccine. Ribavirin possibly useful in severe infections.	None	None
Examples of eploited oncolytic attenuation strategies	E1B55K deletion restricts replication to p53-deficient cells; E3 deletion; E1ACR2 deletion Viral retargeting to receptors expressed only on cancer cells.	Γ34.5 deletion abolishes neurovirulence. UL23 (thymidilate kinase) and ICP6 (ribonucleotide reductase) deletion limit replication to actively dividing cells. Viral retargeting to receptors expressed only on cancer cells.	Not needed – virus does not replicate in healthy human cells and needs actively dividing cells.	Thymidine kinase gene deletions restrict replication to dividing cells.	Use of attenuated vaccine strains (e.g Edmonston strain) as oncolytic agents.	Double mutant strain with mutations in G protein (Q242R) and M protein (L123 W) reportedly oncotropic.	Not needed – virus does not cause significant disease in humans.
Clinical advancement stage	Many phase I or phase I/II clinical trials ongoing for several malignancies including lung, ovarian, pancreatic cancer, glioblastoma and melanoma	Talimogene laherparepvec (®Imlygic, Amgen) approved in the US and EU for metastatic melanoma in the wake of a phase 3 clinical trial (NCT00769704)	Phase I/II clinical trial in primary or recurrent glioblastoma multiforme (NCT01301430)	Phase 3 randomized clinical trial for hepatocellular carcinoma, Pexa-Vec (NCT02562755)	Phase I and II clinical trials with different kinds of tumors, including ovarian cancer, multiple myeloma, and pleural mesothelioma.	Three currently recruiting, open label phase I/II clinical trials for MAGE-A3 expressing solid tumors, non small cell lung cancer and HPV associated tumors (NCT02285816; NCT02879760; NCT03618953)	Several phase I and II clinical trials. One phase 3 clinical trial in association with chemotherapy for head and neck cancer (NCT01166542)

Features described include the genome type and length (which gives an idea of viral capacity as gene therapy vectors), wild-type virus pathogenicity, availability of effective therapies for a “worst case scenario”, the main strategies devised to make viruses selective for cancer cells, and eventually the clinical trial stage reached by viral vectors. ssDNA= single stranded deoxyribonucleic acid; dsDNA= double stranded DNA; ssRNA= single stranded ribonucleic acid; dsRNA= double stranded RNA

Therefore, despite the fact that OVs hold great therapeutic potential, it is clear that they need to be further improved. A better understanding of their in vivo mechanisms of action and pharmacokinetics, as well as a clearer picture of the complex interplay between viruses and host are some of the crucial aspects to be further elucidated to design safer and more effective OVs. Under this respect, different questions remain to be addressed:

1. How “attenuated” should an attenuated OV be? The question may sound trivial, the answer being “attenuated enough to replicate only in cancer cells”. However, the application of this oversimplified principle can have dire consequences, as it does not consider the real complexity of tumors. Many OVs were designed to be able to replicate only in actively dividing cells, for example by deletion of specific genes (like HSV or VACV thymidine kinase and ribonucleotide reductase), according to the idea that cancer cells are actively replicating while healthy cells are not [42]. Unfortunately, many cancer cells within a tumor mass are not undergoing replication. To make things worse, many non-tumoral cells are present in the TME (including macrophages, endothelial cells, lymphocytes, fibroblasts, MDSCs). It has been shown that these cells do not support the replication of OVs designed according to the aforementioned principle and can, therefore, protect malignant cells from viral diffusion [43]. Furthermore, due to the well known cancer heterogeneity, it is hazardous to assume that all cancer cells in all patients will display the same specific molecular characteristic, like mutations within certain onco-suppressor genes and/or overexpression of single pathways [44] and to assume that OV specificity towards cancer cells could rely only on these features [45, 46]. Indeed, evidence demonstrate that the increased susceptibility of cancer cells towards viral replication is the result of variable combinations of alterations mainly in antiviral response and cell cycle regulation pathways. Thus, a more sensible approach for generating OVs, when starting from well-known human pathogens, might be to attenuate them in such a way that they cannot cause the dangerous forms of disease they are associated with (Table 1). In the case of HSV-1, in immunocompetent adults nearly all severe morbidity and mortality is caused by dissemination and replication in neurons, resulting in encephalitis. Therefore, genome modifications that attenuate HSV-1 virulence in neurons might be sufficient to generate a safe OV, despite the fact that the virus retains, at least partially, its ability to replicate in “healthy” fibroblasts or epithelial cells. This feature might even be useful, as it enables the virus to be more effective in the TME, as appears to be in the case of T-VEC.
2. What defines a solid in vitro model to screen for selectivity of OVs towards cancer cells? This question persists despite the fact that, according to what we have suggested above, the focus could be shifted from “unable to replicate in nonmalignant cells” to “unable to replicate in specific target cells relevant for human disease” (neurons in the case of HSV). The problem is associated with the definition itself of “nonmalignant” applied to tissue culture cells. Cell lines, even when incapable of forming tumors once inoculated in immunosuppressed mice, are often immortalized and have very different features from their in vivo counterparts, which can lead to OVs replication in these “healthy” cells. Furthermore, cell lines (including cancer cell lines) are often unpredictable in their susceptibility to viral replication: even viruses with broad cell tropism will occasionally produce very low titers in some cell lines [unpublished observations]. This raises the issue of finding a real “healthy” cell line in which the OV under evaluation is not replicating because the cell line does not have malignant characteristics, rather than because that cell line is characterized by refractoriness to that virus. Primary cells, although technically more demanding, could partially overcome some of these difficulties. On the other hand, organoids derived from malignant and healthy tissue, that are becoming a widely employed in vitro model for several types of studies, would have the further advantage of letting cells grow in a 3D environment, more closely mimicking the in vivo situation [47]. Thus, organoids might represent the best in vitro model to test OV specificity towards cancer cells.
3. What is the most appropriate animal model to test safety and efficacy of OVs? Most studies so far relied on SCID or nude mice, which are a readily available model in which murine or human cancer cell lines can thrive because of immunosuppression [48]. Still, this feature (especially the absence of a competent T cell response) profoundly alters the mechanism of action of OVs. In these models, direct oncolysis by the virus could actually be the main effector mechanism that prolongs survival of the animals, which is probably not what happens in human patients. On the other hand, the use of immunocompetent mice could partially overcome this difficulty. However, the differences between the murine and human immune systems are still a hurdle, especially when the ability of the OV of influencing the immune response against cancer cells is under evaluation. Humanized mice, i.e.completely immunodeficient mice which receive a human hematopoietic stem cells (HSC) transplant plus human fetal thymus and liver tissue to guarantee T cell maturation, could provide an answer to this difficult problem [49].
4. What is the exact role of the host immune system/OV interaction in determining the success of virotherapy for tumors? Some investigators argue that the immune system has a deleterious effect, since it could wipe away the OV, especially if deriving from a human pathogen widely present in the population, before it can kill a sufficient number of cancer cells. As a consequence, efforts were spent on concealing the virus from the immune system, or on using viruses for which a preexisting immunity in the general population is unlikely [50]. However, this concept is clearly rooted in the older paradigm of OVs directly killing cancer cells rather than in the recent idea of OVs as tools for immunotherapy. Indeed, recent data strongly suggest that the release of danger signals and inflammation due to OV replication, along with immune system activation against infected cells, account for an important part of the antitumoral potential of the OV itself [51]. Furthermore, in the case of T-VEC, no differences in therapeutic effects were observed between HSV-1 seronegative and seropositive patients. Recently, a study using a mouse model of melanoma treated with the paramyxovirus NDV showed enhanced antitumoral activity in mice with preexisting immunity to NDV [52]. Finally, after intratumoral treatment with T-VEC, it was observed that uninjected lesions, including some visceral metastases, underwent regression. In a recent clinical trial, regression was heralded by activated CD8+ lymphocytes infiltration and was enhanced by checkpoint inhibitors [53]. Such a pattern is consistent with an immune response elicited by viral injection in multiple accessible lesions but effective also against uninjected lesions in which the virus was not detectable.
5. What is the best route of administration for an OV? It is often stated that an ideal OV should be systemically injectable, for some good reasons: essentially, the possibility to infect both primary tumor and metastases, and the fact that this route is relatively non-invasive and injections can be frequently repeated [54]. However, although some OVs (VACV, T3D orthoreovirus, H-1 parvovirus), were administered intravenously to human patients without severe side effects, the most used route is the local (intratumoral) injection [55]. This is the case also for the only approved OV, T-VEC. Intratumoral delivery is usually chosen because of safety concerns after intravenous injection, or, especially in the case of HSV-1, to minimize the chance that preexisting circulating antibodies might neutralize the virus before it reaches its target, as discussed above [56]. Nevertheless, as mentioned above, in the case of T-VEC, despite the intratumoral injection, uninjected lesions and visceral metastases displayed a regression, likely due to the immune response elicited by the virus [41].
6. Viruses have an important feature, which makes them particularly appealing as cancer therapeutics: they are not just cancer cell killers or a proinflammatory stimulus, but they can also serve as platforms for the delivery/expression of transgenes. This feature allows the development of OVs “armed” with therapeutic genes, some of which are already under evaluation in clinical trials. One example is again T-VEC that, in addition to specific mutations within viral genes, carries two copies of the human granulocyte-monocyte colony stimulating factor (hGM-CSF) encoding sequence, under the transcriptional control of the human CMV immediate early promoter [57]. However, most of these engineered viruses only express a single immunostimulatory cytokine or ligand [58, 59]. As a result, these OVs do not exploit the wealth of information that was recently accumulated on cancer immunology and the TME [60], and may even be outdated. For instance, recent comprehensive reviews cast a dubious light on the usefulness of hGM-CSF in cancer immunotherapy [61]. The issue with cancer immunotherapy is not simply boosting a “sleeping” immune response, but the fact that cancer cells actively use immunosuppressive mechanisms and recruit tolerogenic cells. OVs could be used to locally deliver high and constant concentrations of single-chain antibodies or other protein ligands that disrupt those immunosuppressive features. Under this respect, many different strategies can be devised, including the expression of enzymes that degrade the abundant extracellular matrix present in some tumors (desmoplastic reaction) or of dominant negative forms of immunosuppressive cytokines (for example TGF-β) [62].
7. More specifically, could there be space for engineered OVs expressing checkpoint inhibitors, as single chain antibodies, whole antibodies, or proteins that may have the same function? Potent immune checkpoint inhibitors (CKIs) which are delivered systemically are already available, and most investigators are focusing on synergism between existing checkpoint inhibitors and OVs [63]. However, CKIs expressed as therapeutic genes by OVs would probably have the advantage of a prolonged and localized delivery in the TME, which might, in principle, avoid the autoimmune side effects usually associated with systemic CKIs.
8. Is there the possibility of a more extensive cell-specific reprogramming of viruses? Ideally, once an OV reaches the TME, it should produce different effects in different types of cells. Of course, it should replicate in cancer cells and cause their death while sparing surrounding normal tissue and/or non causing severe diseases. However there could be further nuances. For example, an OV could be designed to specifically trigger a Th1 phenotype in infected macrophages or to replicate also in cancer associated fibroblasts or endothelial cells that might become a more persistent “factory” of therapeutic gene products. Transgenes under the transcriptional control of cell-specific promoter might serve to this end. While “promoter retargeting” has been explored to enhance viral replication in cancer cells, such an approach to the diversity of the TME has not been investigated yet, at least to our knowledge.

## Conclusions

Cancer immunotherapy is establishing new paradigms in the treatment of advanced stage solid malignancies. Together with immune checkpoint inhibitors, OVs are increasingly recognized as a promising therapeutic tool in this field. The use of OVs on patients has become a clinical reality in the case of talimogene laherparepvec, also known as T-VEC, for metastatic melanoma, and recent clinical trials strongly suggest that the combination of talimogene with CKIs could be particularly effective in this setting [23, 53]. Despite this successes, OV treatment of cancers other than melanoma, which is usually considered a very immunogenic tumor, has given limited clinical results [64, 65]. Several recent studies have been trying to characterize the antitumoral immune response after OV therapy both in mouse models and in patients enrolled in clinical trials [53, 66]. However, current basic and translational research on OVs is mainly focused on safety (which, however, has never been a real issue over decades of clinical trials), on various combinations of OVs with chemo and radiotherapy or CKIs, and on the quest for “exotic” non-human viruses, whose ability **t**o infect and lyse a significant number of human cancer cells in vivo remains questionable [67].

The feeling that OVs are not being exploited to their potential is increased by the lack of new ideas on the use of OVs as platforms to express factors aimed at increasing their killing ability and the immunomodulatory effect. Under this respect, there have not been many novelties in the last years, at least to our knowledge. Indeed, in the field of OVs, viral engineering has been mainly employed for the attenuation of the human pathogen under evaluation, for its transcriptional [68] or receptorial [69] retargeting, for the expression of suicide genes or single immunostimulatory cytokines (as in the case of talimogene). Thus, there is wide space for the design of innovative OVs to better achieve, for instance, TME modulation. The ideal candidate would be a large dsDNA virus that can allow the insertion of multiple transgenes within its genome, without losing its ability to replicate in and kill cancer cells, and for which “robust” gene editing techniques are available.

**Fig. 1:**
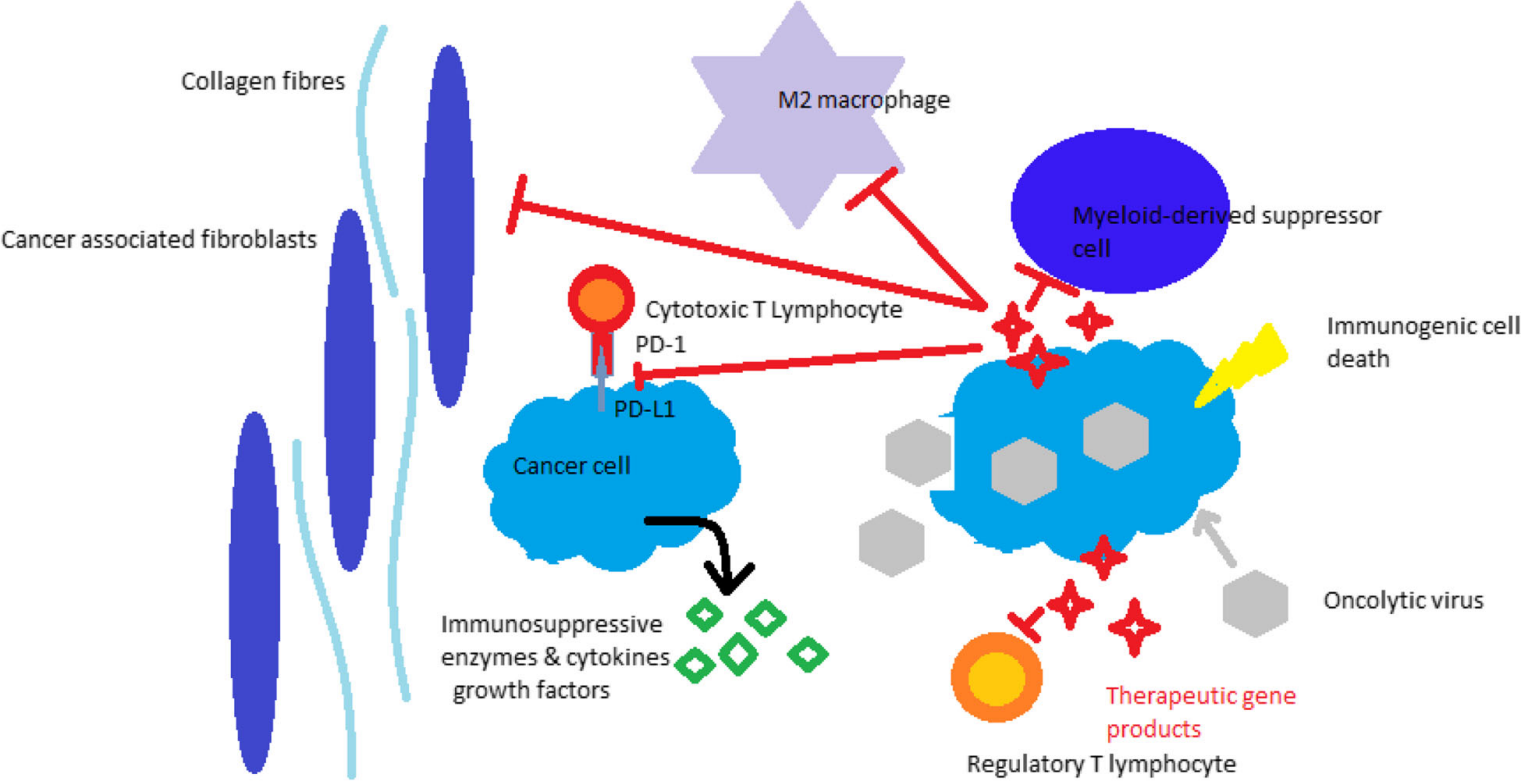
Schematic representation of cancer cells (cyan) in their immunosuppressive microenvironment, which they shape by secreting cytokines and growth factors. Immune checkpoint molecules and immunosuppressive enzymes released in the milieu inhibit cytotoxic T lymphocytes. Infection of cancer cells by an oncolytic virus (OV, gray) disrupts the immunosuppressive features of the microenvironment by triggering immunogenic cell death and releasing proinflammatory substances. OVs can also be armed with therapeutic genes targeting non malignant cells that support tumor growth and immune escape, such as cancer associated fibroblasts, M2 macrophages, myeloid derived suppressor cells (MDSCs), and regulatory T lymphocytes

Finally, the big challenge that OVs are facing is the therapy of immunologically “cold” tumors which are usually failing to respond to checkpoint inhibitors due to the absence of a lymphocyte infiltrate. The presence of a virus (especially of a replication competent virus) can profoundly alter the TME by enhancing the immune cell infiltrate and generating proinflammatory cues. Is this enough to make cold tumors sensitive to immune checkpoint inhibition? It must be considered that these tumors often display an immunosuppressive immune cell infiltrate or a fibrotic microenvironment, which could protect malignant cells from the immunogenic stimuli provided by the virus. Armed OVs might be once again the solution to this problem. Indeed, therapeutic gene products, released at high concentrations in situ by infected cells, could synergize with OVs by killing immunosuppressive cells or inhibiting their activity. Furthermore, OVs could be engineered to express enzymes that degrade the fibrotic extracellular matrix, thus helping to tackle very “difficult” tumor microenvironments [Fig. 1].

## Abbreviations

CKI: Checkpoint Inhibitors
CTLA-4: Cytotoxic T Lymphocyte Antigen 4
hGM-CSF: human Granulocyte Monocyte Colony Stimulating Factor
HSV: Herpes Simplex Virus
OV: Oncolytic Virus
PD-1: Programmed Death 1
PD-L1: Programmed Death Ligand 1
TME: Tumor Microenvironment
